# Modeling and Fault Detection of Brushless Direct Current Motor by Deep Learning Sensor Data Fusion

**DOI:** 10.3390/s22093516

**Published:** 2022-05-05

**Authors:** Priscile Suawa, Tenia Meisel, Marcel Jongmanns, Michael Huebner, Marc Reichenbach

**Affiliations:** 1 Department of Computer Engineering, Brandenburg University of Technology Cottbus-Senftenberg, 03046 Cottbus, Germany; michael.huebner@b-tu.de (M.H.); marc.reichenbach@b-tu.de (M.R.); 2 Fraunhofer Institute for Photonic Microsystems, 01109 Dresden, Germany; tenia.meisel@ipms.fraunhofer.de (T.M.); marcel.jongmanns@ipms.fraunhofer.de (M.J.)

**Keywords:** accelerometer, brushless direct current motor, deep convolutional neural networks, deep learning sensor fusion, faults detection, long short-term memory, microphone

## Abstract

Only with new sensor concepts in a network, which go far beyond what the current state-of-the-art can offer, can current and future requirements for flexibility, safety, and security be met. The combination of data from many sensors allows a richer representation of the observed phenomenon, e.g., system degradation, which can facilitate analysis and decision-making processes. This work addresses the topic of predictive maintenance by exploiting sensor data fusion and artificial intelligence-based analysis. With a dataset such as vibration and sound from sensors, we focus on studying paradigms that orchestrate the most optimal combination of sensors with deep learning sensor fusion algorithms to enable predictive maintenance. In our experimental setup, we used raw data obtained from two sensors, a microphone, and an accelerometer installed on a brushless direct current (BLDC) motor. The data from each sensor were processed individually and, in a second step, merged to create a solid base for analysis. To diagnose BLDC motor faults, this work proposes to use data-level sensor fusion with deep learning methods such as deep convolutional neural networks (DCNNs) for their ability to automatically extract relevant information from the input data, the long short-term memory method (LSTM), and convolutional long short-term memory (CNN-LSTM), a combination of the two previous methods. The results show that in our setup, sound signals outperform vibrations when used individually for training. However, without any feature selection/extraction step, the accuracy of the models improves with data fusion and reaches 98.8%, 93.5%, and 73.6% for the DCNN, CNN-LSTM, and LSTM methods, respectively, 98.8% being a performance that, according to our reading, has never been reached in the analysis of the faults of a BLDC motor without first going through the extraction of the characteristics and their fusion by traditional methods. These results show that it is possible to work with raw data from multiple sensors and achieve good results using deep learning methods without spending time and resources on selecting appropriate features to extract and methods to use for feature extraction and data fusion.

## 1. Introduction

Cyber-physical systems directly support the agenda of Industry 4.0, which is a crucial driver of the worldwide economy and growth in industrial production, industrial automation, and related logistics. The leading industries in this area need novel solutions to develop energy-efficient, highly flexible, robust, safe, and secure systems for the next generation of factories in the age of Industry 4.0. The key elements here are to increase efficiency, decrease time to market, and enhance flexibility. Industry 4.0 achieves very promising improvements for production processes, including reducing cost, improving quality, and manufacturing almost personalized products. All these benefits are related to much more data about the status of a production process being available to react to a current situation or even predict a status in the future. Reducing production costs can also be considered as a means to increase the sustainability of the production process. In other words, if all the additional data provided by Industry 4.0 are available, the break-and-replace cycles that can lead to high costs due to production downtime will be reduced tremendously. An approach to predicting maintenance will save resources such as personal costs and material, which may not be used for further production anymore (e.g., in metal, chemical, or biological production processes). One step ahead in novel production environments is to predict maintenance cycles of machines so precisely that the machine downtime can be scheduled optimally during time slots, where a production loss does not lead to trouble in the overall production processes in a plant. Many industries still use time-based maintenance, which is maintenance performed at fixed time intervals according to a schedule. It is costly because it could lead to unnecessary maintenance when the machines do not encounter any problem [1].

The challenge is to have resilient industries that implement predictive maintenance (PM). This type of maintenance is scheduled according to prediction algorithms that can determine the expected time of future damage to the machine. Therefore, it tries to achieve the best trade-off between executing the maintenance too early (which could lead to losses by replacing parts that are still functional) and too late (which could also lead to time losses due to unexpected machine downtimes). To ensure success, it is preferable to have living predictive models, i.e., models that will be able to adapt to changes in the environment using real-time sensory data. According to [2], the appropriate technology to propel the performance of such models would be data fusion, which facilitates the flow of information from raw sensory data to high-level understanding and information. However, the choice of machine learning algorithms that will be used to train the models with the raw data must be careful. In accordance with [3], deep learning, which is a subdivision of artificial intelligence, is defined as a set of multi-layered network-building algorithms that allow them to process raw data and extract certain patterns to perform complex and intelligent tasks.

This paper addresses the PM topic by artificial-intelligence-based analysis of signals measured by sensors connected to brushless direct current (BLDC) motors. The scientific literature counts numerous papers about fault diagnosis on applications with BLDC motors associated with different types of sensor data and different ways to combine them, including data-level, feature-level, and decision-level combinations; they deal with input data patterns of distinct types. To the best of our knowledge, all the state-of-the-art works on PM of BLDC have employed feature-level fusion [4,5,6,7]. The methods presented require extensive knowledge of the application domain and signal processing. They usually yield good precision in fault detection and diagnosis. For example, Shifat and Hur reached around 98% accuracy, with feature-level fusion [4].

In contrast to this approach, data-level fusion is more generic and requires less expertise in signal processing. The reduced complexity of signal processing algorithms often leads also to a reduced performance requirement in embedded systems, which is crucial when the algorithms are implemented on a local sensor node. However, sensor values come with disturbances or even interruptions, which can be handled with traditional signal processing algorithms. The trade-off is to find a balance between the traditional algorithms and the AI methods and which data are used as input data for the AI. In general, all methods must deal with raw data imperfection, no matter how complex the data. The challenges we address are showing that data-level fusion leads to a benefit with a similar structure of the collected data, which deep learning algorithm is a promising candidate for sensor-data-level fusion, and how a general paradigm can be extracted from our investigations and experimental setup.

The main task in PM is the recognition of the health conditions of a system [8]. To explore different types of sensory data and their combination at the data level, we built an experimental setup (described in Section 5.1) that allowed us to construct a new dataset with vibration and sound data obtained from sensors connected to a BLDC motor. This new dataset was used to perform the detection of different loads and weights that were used to emulate real tools such as drills and cutting heads that are mounted on a BLDC motor. With this setup, three methods were evaluated and compared: deep convolutional neural networks (DCNNs), the long short-term memory method (LSTM), and convolutional long short-term memory (CNN-LSTM). Our main results are as follows:

- In our configuration, the sound signals performed much better than the vibration signals when it came to assessing the condition of the machine, with an accuracy of 96.8% compared to 71.1% with vibration for the DCNN model.
- Using the data from both signals with deep learning sensor fusion, the three models DCNN, CNN-LSTM, and LSTM achieved 98.8%, 93.5%, and 73.6% accuracy, respectively.
- The DCNN algorithm is the most promising deep learning sensor fusion algorithm for BLDC motor fault diagnosis.

With these results, we show that our new dataset was well constructed and achieved good results, such as those obtained with the public dataset mentioned in Section 6. We also show that a robust algorithm can train strong models with single sensor data to perform an accurate detection task. Finally, our experiments show that good results can be achieved with less effort, resources, and time when sensor fusion is performed at the data level with an appropriate deep learning sensor fusion method.

The remainder of this article is structured as follows: Section 2 reviews related work. A background on predictive maintenance and sensor fusion is provided in Section 3. The description of the proposed approach is presented in Section 4. Section 5 describes the setup and the data collected from it. The different methods explored are presented in Section 6. Section 7 provides the details of the training processes. The results and discussion are presented in Section 8. Section 9 concludes and presents the future work.

## 2. Related Works

Various industrial applications of sensor fusion can be found in the literature, including planning of production processes [9,10], identification of different parts [11], monitoring the melting process of blast furnaces [12], and PM [13], among others. To present a broad review of the different ways sensor fusion is implemented, this section is not only limited to the application of PM to BLDC motors and its tools.

Regarding the BLDC motor’s fault diagnosis, the existing state-of-the-art works are based on feature-level sensor fusion. In [14], frequency-domain features were extracted, then fused from four sensors (one vibration sensor and three current sensors), and a back-propagation neural network (BPNN) was used to classify six health states. The authors showed that using a single current signal, the model achieved an accuracy of about 93.5%, compared to 98.3% when using the fusion of three phases of current signals. A feature-level sensor fusion of a vibration sensor and a current sensor was performed in [4,6,7]. In [6], a discrete wavelet transform was applied to extract sensory features, then feature reduction was performed by orthogonal fuzzy neighborhood discriminative analysis (OFNDA), and a recurrent neural network (RNN) was used to classify four bearing states. In [4], several features were extracted from the sensor signals in the time and frequency domains. The most relevant features were then selected based on monotonicity and correlation, and then, PCA was used for feature reduction. An artificial neural network (ANN) was used to classify three health states. The authors in [7] studied the combination of three techniques, fast kurtogram (FK), autogram, and motor current signature analysis (MCSA), to classify three states.

The authors in [15] examined the single sensor case and all the sensor fusion cases (data, feature, and decision fusion) of four sensors. The DCNN, support vector machine (SVM), and back-propagation neural network (BPNN) were used for diagnosing a planetary gearbox. The results indicated that using a single vibration signal, the DCNN, BPNN, and SVM models achieved an accuracy of 81.45%, 42.56%, and 45.11%, respectively, compared to 99.8%, 53.28%, and 51.62% using a data-level fusion of four signals: the instantaneous angular rate (IAS) signal, vibration, acoustic, and current signals. In addition, the authors presented various results obtained with feature-level fusion. A multi-scale CNN-LSTM model was proposed in [16] to diagnose a rolling bearing using raw accelerometer signals. Ten classes of the bearing health states were considered (a normal state and nine faulty states). The results showed that the proposed method was able not only to achieve an average accuracy of 98.46%, but also to outperform some advanced intelligent algorithms based on prior knowledge such as LSTM and SVM.

The authors in [17] performed a multi-sensor feature fusion to assess the health conditions of rotating machinery. The autoencoder (SAE) neural network for feature fusion and the deep belief network (DBN) for training demonstrated that the fusion of four vibration signals recorded under different running speeds achieved higher accuracy compared to the case of a single vibration signal. The authors in [18] developed a sensor-fusion-based in-depth feature learning approach to identify the gear crack severity. Three stacked autoencoders were used to fuse features extracted manually from three sensors. The fused features were then presented to the classifier, where four classes of crack severity were considered. The experiments showed that the developed approach achieved an accuracy of 90.4% with the fusion of the three sensors’ signals, compared to about 80% with each signal. A deep belief network was applied in [19] to predict the cutter wear out using three sensors.

The authors in [9] proposed a multi-stage deep learning classifier to determine the other production processes based on the quality of products. At first, stacked LSTM autoencoders were used to extract the features from the sensor signals, and then, a deep feed forward neural network was used for three-class classification. In [12], a seven-class RNN classifier was used to classify the variation trend of silicon content to monitor the blast furnace. The inputs of the classifier were generated through a developed multi-level feature fusion. Three feature sets were fused: basic features from the process, statistical features, and abstract features learned through stacked denoising autoencoders (SDAEs). Acoustic emission (AE) is an effective non-destructive testing method used to detect failures. To recognize and predict coal rock burst hazards, the authors of [20] proposed a new multi-resolution feature fusion SVM (MRFF-SVM) recognition approach. Three improved processes were included in the proposed approach: the coiflet wavelet transform (CWT) to split AE waveforms into multiple perspectives for feature vector extraction, a multi-resolution feature fusion (MRFF) method to merge these vectors into an improved MRFF feature vector, and support vector machines (SVMs) to identify coal rock bursts. In [21], the authors presented a fusion of vibration and acoustic emission features for tool condition monitoring systems in metal cutting processes. Time domain, frequency domain, and time–frequency domain features were extracted and merged to classify each tool condition using a machine learning classifier.

The authors in [22] proposed a method to implement a sensor fusion with heterogeneous data. The technique was called multi-layer attribute-based conflict-reducing observation (MACRO), and it aims to reduce the effect of conflicting input data on the fusion result. Unlike the existing BLDC-related works that were mentioned above [4,5,6,7], our work is not based on the feature-level sensor fusion. Similar to [15], our work is based on data-level sensor fusion. It relies entirely on the capability of a deep learning model to extract/fuse features from the combined sensor signals and make the classification decision, as shown in the subsequent sections.

## 3. Background

### 3.1. Predictive Maintenance

The concept of predictive maintenance could be defined as a continuous monitoring approach to anticipate system failures, which will lead to maximizing the time interval between consecutive maintenance tasks and reducing the overall costs of production [23]. There are three main types of maintenance approaches capable of monitoring the condition of equipment for diagnostic and prognostic purposes: statistical, artificial intelligence, and model-based approaches. Since model-based approaches rely on mechanical knowledge and the theory of the equipment to be monitored and statistical approaches require mathematical expertise, artificial intelligence approaches in the field of predictive maintenance are becoming increasingly popular. With the increasing amount of industrial data in the fourth industrial revolution, current works applied deep learning solutions such as the autoencoder, convolutional neural network, deep belief network, and others for predictive maintenance tasks, as reported in [24]. Bearings, blades, motors, valves, gears, and cutting tools are key components commonly monitored. Common types of failures detected include tool imbalances, fatigue, abrasive and corrosive wear, friction, defects, and leakage detection, among others [25].

Industrial use cases face two major challenges: their behavior and the variability of their data. Even equipment with the same specifications is likely to encounter these problems, due to mechanical tolerances, assembly adjustments, environmental variations, etc. The above factors make it difficult to reuse PM models on different machines and equipment. Other significant challenges include collecting quality data, especially failure data, and performing appropriate pre-treatment and feature engineering to obtain a dataset that represents the problem [25].

The shortcomings of PM include the fact that systems can lead to false maintenance requests, due to the misinterpretation of the data; predictive analysis may not take into account contextual information, such as the age of the equipment or the weather. However, PM systems need to combine several properties to ensure a minimum accuracy, including robustness, adaptability, identifiability of multiple faults, etc. To this end, the use of different sensors and the fusion of their data for equipment monitoring are a major asset as this allows a diversified representation of information describing the environment.

### 3.2. Sensor Fusion

Sensor data are gradually driving the Internet of Things (IoT), with devices performing actions measured based on everything from sound, vibration, speed, temperature, and more. Each datum is to be captured, sent, and finally used, which poses a constant challenge for companies. Understanding how to process sensor data can help developers and decision-makers obtain the most from this powerful information. Data from one or more sensors can be used individually or combined to assess the condition of a machine. Sensor fusion refers to integrating data from two or more sensors to achieve a more consistent representation of the observed phenomenon and reducing individual sensor limitations [14]. The main advantages of sensor fusion include increased reliability, robustness, and accuracy, extending the system coverage, and reducing uncertainty [26]. According to the level at which the fusion occurs, three main categories of data fusion techniques can be defined:

- Data-level fusion: This method of fusion combines raw data from different sensors to build a data sample. The fused sensors’ data structures should be similar, and synchronization between sensory signals should be achieved before fusing [14]. This fusion has higher requirements for storage, communication, and computation resources than the other two categories of sensor fusion. However, since the data-level fusion occurs in an early stage of the processing chain, it does not cause any loss of the raw data’s inherent details, which helps to achieve high accuracy in classification tasks. Furthermore, it does not rely on signal analysis, which requires knowledge of signal processing. These two advantages are beneficial in handling complex fault diagnosis tasks [16].
- Feature-level fusion: Here, the features are extracted from individual sensors and then combined in one feature sample that can be input to a pattern recognition model [27]. This fusion level can be advantageous when the sensors to be fused have different data formats. However, for feature extraction, some transformations of the raw sensor signals are usually needed, e.g., fast Fourier transform and discrete wavelet transform. Furthermore, it is essential to implement feature selection and reduction techniques, e.g., principal component analysis (PCA), to reduce the dimension of the fused feature sample [27]. These processes require knowledge of signal processing and mathematics, and they are also time-consuming in terms of algorithmic complexity, which leads to increased latency on computing systems [16].
- Decision-level fusion: This fusion involves separate decision-making for each sensor and then combining those decisions to provide the final decision. Due to the presence of several separate decisions, this category is the most resilient compared to the previous two methods when it comes to individual sensor data recognition such as in healthcare applications [26]. However, considering the detection and identification tasks, the improvement gained by this fusion is not significant compared to the other two fusion methods reported above because each decision is only based on the information provided by one sensor [28].

## 4. Description of the Proposed Approach

Fault detection is an important task to ensure the proper functioning and stability of an industrial system. When present in equipment, motors are the main components that allow the equipment to operate. However, it is very difficult to analyze the condition of motors because they are usually installed in a hostile environment, generating noise-filled data. The work presented in this paper focuses on BLDC motors. An experimental setup that can be used as a basic model for small motor faults or speed measurements was designed to record data in a real environment that were used for training. The investigations carried out allowed us to draw a comparative figure between the approach used so far for fault detection of a BLDC motor and the approach proposed in this work.

**Figure 1:**
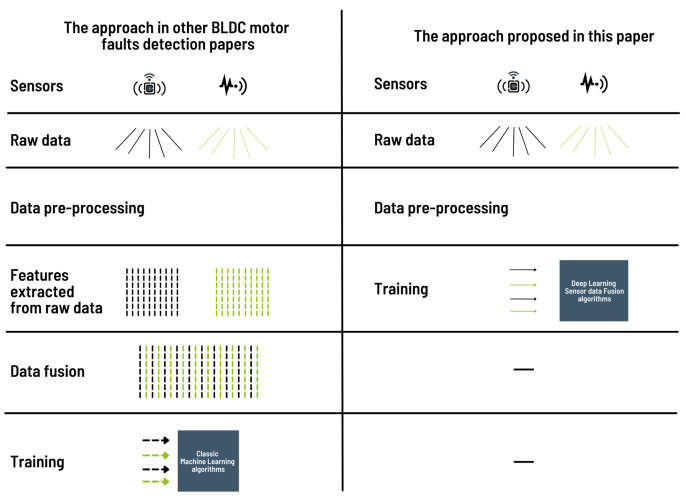
Comparison of the approach used so far with the proposed approach for the BLDC motor fault detection task.

Figure 1 shows on the left the approach used by the authors as presented in the Related Work Section and on the right the approach proposed in this paper. The former contains four steps, whereas we propose a two-step approach. The first step when the raw dataset is available is the same in each approach. This is the preprocessing of the data, which results in the removal of noisy and redundant data. After this step, we propose to move directly to training the detection model using deep learning methods or algorithms. Some of these methods can extract important information, merge it, and then use it for training. This multi-faceted approach combines the last three steps of the approach used so far by the authors who have explored fault detection in a BLDC engine. This avoids the need to worry about the selection/extraction of information and the choice of fusion algorithms, which often require technical skills of the studied equipment to know the type of characteristics to be extracted, but also skills in signal analysis. However, most of the time, machine learning engineers have to deal with datasets without having the technical skills mentioned above. The approach we propose saves time because it is summarized in two steps that avoid machine learning researchers investing in understanding the technical details related to the studied domain.

The work carried out set up an experimental system for the collection of real data, which were used to train by three deep learning methods, namely the DCNN, LSTM, and CNN-LSTM.

## 5. BLDC Motor: Setup and Data

**Figure 2:**
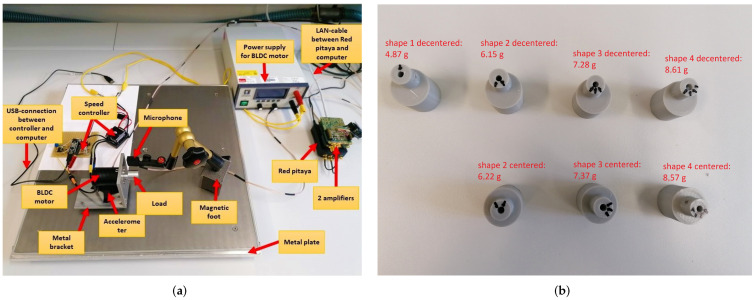
Experimental setup. (**a**) Setup. (**b**) Loads mounted on the motor.

As mentioned earlier, in this work, we used a deep learning sensor data fusion for identifying different tools attached to a BLDC motor by analyzing sound and vibration. The experimental setup is presented in Figure 2a.

### 5.1. Experimental Setup

The BLDC motor was attached to a bracket, which was fixed to a metal plate by a suspension. The speed of the motor can be configured by the speed controller. The motor was connected to a power supply, and a USB cable connected the controller and the computer to display the speed of rotation. A microphone and an accelerometer were used to measure sound and vibration, respectively. A hydraulically clamped magnetic stand was used to place the microphone near the loads. The accelerometer was glued to the elastic support of the motor. Both the microphone and the accelerometer were connected to the Redpitaya-Board via an amplifier. The Redpitaya-Board is a small PC and uses a Xilinx Zynq 7010 System-on-Chip. This system-on-chip combines a dual-core ARM Cortex-A9, which runs a Linux distribution, and an FPGA, used for fast data acquisition and artificial intelligence acceleration. The board was deployed to acquire amplified signals via an analog-to-digital converter (ADC). FPGAs allow close hardware collaboration and hardware acceleration of algorithms. The board was needed because of the high sampling rates. The board was used to collect the data and transmit the acquired data via Ethernet to a host PC. In the next steps, the algorithms was implemented on the FPGA and the processor on the system-on-chip with an HW/SW design process, where the artificial intelligence algorithms specifically benefit from the acceleration offered by the FPGA.

Both the microphone and the accelerometer use a sampling rate of 1,953,125 Hz. During recording, the data were stored in packets of 16,384 samples. The packets were cached so that the recording frequency remained between 15 and 20 Hz. The Redpitaya-Board was connected via a local network to the host computer, where the data were finally stored. The data were recorded in simple text files.

### 5.2. Data

In this study, we chose a microphone and an accelerometer as sensors for fault diagnosis of the BLDC motor:

- A microphone is an acoustic electrical transducer or sensor that detects and converts sound pulses into electrical signals. In machine diagnostics, the acoustic signal is one of the most important sources of information. Most of the events that are important for the diagnosis of machines and processes can be detected and evaluated efficiently using an acoustic signal. It requires significant computing capacity and significantly higher sampling rates for signal processing [29].
- An accelerometer is an electromechanical instrument that monitors acceleration forces. There are several types of sensors that can detect the magnitude and direction of the acceleration as a vector quantity and can be used to detect position, shock, etc. [30]. Accelerometers measure vibrations in rotating equipment. They are the most commonly used data sensors for fault detection and condition monitoring of rotating machinery, as they are easy to handle and process [31].

These two sensors were selected for the various reasons mentioned above. Since errors in industrial operations are unpredictable, for example the electrical and mechanical signatures of a motor can vary greatly depending on the degree of stress to which it is subjected [4], an effective condition monitoring system requires multi-sensor data. Multi-sensor data involve sensor fusion, which suffers from two main challenges when used in combination with complex tasks such as fault diagnosis. These are the selection of the fusion level and the extraction of features from multi-sensor data, as the sensor data evolve in different ways. These challenges are present due to increasing uncertainties in measurements, increasing conflicts, noise, increasing data dimensions, etc. [15]. In addition, data from one sensor may indicate abnormal behavior, while the other may show no detectable change in trend.

The experiments carried out in this work produced a large amount of data. Therefore, taking into account the various concerns mentioned above, we developed fault diagnosis models based on deep learning data fusion algorithms that can automatically merge the input data and extract relevant features [15].

**Figure 3:**
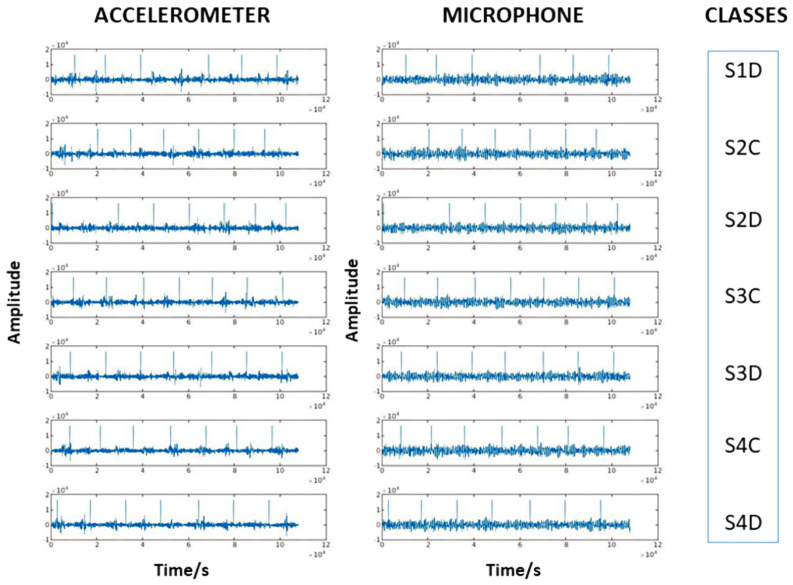
Sample of recorded accelerometer and microphone signals for each class.

The experimental setup includes seven different tools to distinguish: Shape 1 decentered (S1D), Shapes 2, 3, and 4 centered and decentered (S2C, S2D, S3C, S3D, S4C, S4D; see Figure 2b). The different weights with centered/decentered characteristics were considered in this work as drivers of different types of faults. In each experiment, only one of them was mounted on the motor and examined. During the experiment, the speed of the motor varied between 4000, 6000, 8000, 10,000, 12,000, and 14,000 revolutions per minute (rpm). Table 1 provides a detailed description of the data set. Six signals per data type were acquired. The six signals correspond to the different speeds set after each run of the system. Each signal was captured for five minutes. Figure 3 shows sample signals captured from the accelerometer and microphone for each class.

## 6. Methods

### 6.1. Data Processing

**Figure 4:**
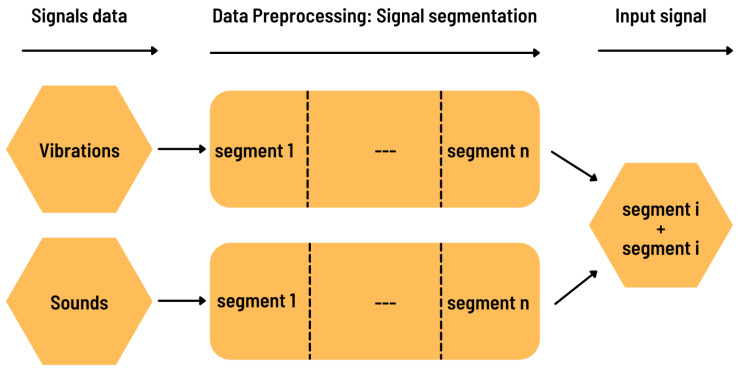
Preprocessing.

The preprocessing step (see Figure 4) was based on Luyang et al. [15]. After removing noises from the collected signals, they were divided into small segments of 1000 points each. At the end of the segmentation step, the microphone and accelerometer signal segments were combined to form the input vectors of the model. Each input vector was a vector of 2000 points.

### 6.2. Deep Learning Sensor Fusion

Often considered as an improvement of neural networks, deep learning is a component of artificial intelligence and machine learning that attempts to mimic human thinking. Deep learning algorithms create multi-layered networks that process raw data and extract patterns used to perform complex, intelligent tasks by analyzing them. In the field of deep learning, there are many different types of algorithms. Each technique is unique and is tailored to certain applications, where the goal is to achieve the best possible performance. There has been a notable increase in the amount of research associated with deep learning sensor fusion algorithms, with convolutional and recurrent neural networks (CNN and RNN) being among the most widely used [3]. In this work, three deep learning methods were investigated, namely: the DCNN for its ability to automatically extract important features from the raw input data, combine them through its multiple layers, and thus, result in an accurate model; LSTM because it is a method that deals with long-term relationships between data, allowing a decision to be made at a given point in time based on a set of information stored at previous times [3,25]. This is an advantage for predictive maintenance of equipment, as it allows a decision to be made at a given time on the state of an item of equipment based on the history of events that have occurred during its operation. Since the approach used in this paper involves passing raw data into the inputs of the different methods, the CNN-LSTM method was studied here to assess whether the addition of a convolutional layer to the LSTM could improve its performance since the LSTM method does not incorporate layers that extract features from the raw data.

#### 6.2.1. Deep Convolutional Neural Network

**Figure 5:**
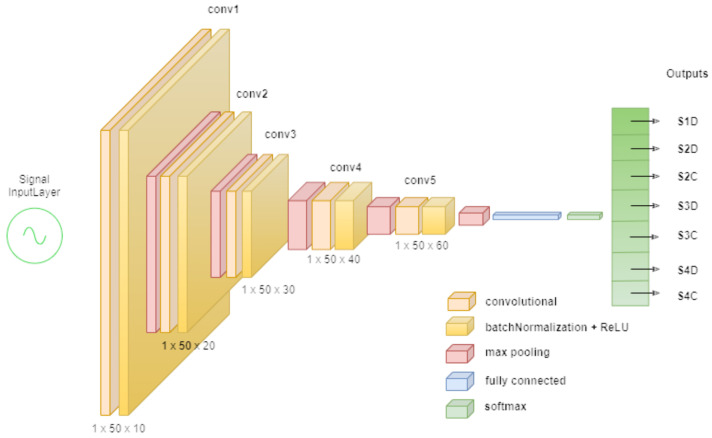
DCNN architecture (S: shape, D: decentered, C: centered).

The architecture of the DCNN model used in this work is shown in Figure 5. It is based on a classical CNN and takes a concatenation of raw vibration and sound signals as the input and passes them through a set of convolutional filters, each of which activates certain features of the signal. This process on the convolutional filters is repeated on the five convolutional layers in this architecture. To speed up network training and reduce sensitivity to initialization, batch normalization layers are used between convolutional layers and non-linear activation layers (ReLU). We used pooling layers to simplify the output and reduce the number of parameters the network had to learn by performing non-linear downsampling. This architecture was used to train on data from our setup, with the following training options, minibatch size: 2000, max epochs: 10, optimizer: Adam [32], learning rate: 0.001.

#### 6.2.2. Long Short-Term Memory

**Figure 6:**
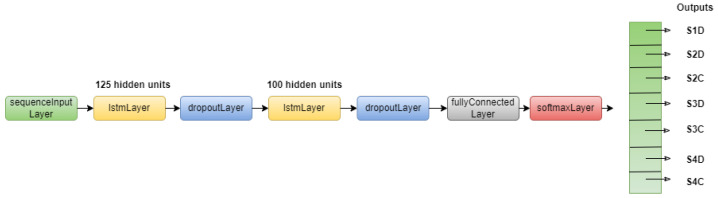
LSTM architecture.

The LSTM architecture presented in Figure 6 is the one used for our experiment. This architecture is built with an input layer, which also takes as input the concatenation of signals like the previous method. The input layer is followed by two LSTM layers that learn long-term dependencies, each of which is followed by a dropout layer to prevent overfitting. While LSTM can handle long-term dependencies, it still needs to go deep to learn a high-level representation and model complex dynamics [33]. The number of hidden units represents the amount of information stored between the time steps. The last two layers are used for the classification. This architecture is used with the following training options: minibatch size: 2000, max epochs: 10, optimizer: Adam [32], learning rate: 0.001, gradient threshold: 1.

#### 6.2.3. Convolutional Long Short-Term Memory

**Figure 7:**
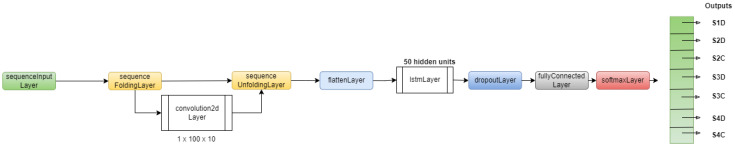
CNN-LSTM architecture.

The architecture of the CNN-LSTM trained in this work is shown in Figure 7. This architecture also takes as input the concatenation of the two types of raw signals. To automatically learn effective features suitable for tool health detection, a convolutional layer is used to perform feature extraction on the concatenation of the two input signals. To perform the convolutional operations on each time step independently, a sequence folding layer is included before the convolutional layer to convert the sequence input into an array. As the LSTM layers expect a sequence input, a sequence unfolding layer and a flattening layer are used between the convolutional layers and the LSTM layer to restore the sequence structure and reshape the output of the convolutional layers into a sequence of features. To train on our data, we used this architecture, with the following training options: minibatch size: 1000, maximum epochs: 100, optimizer: Adam [32], learning rate: 0.001, gradient threshold: infinite.

## 7. Training

The training of the DCNN model started with an open-access data set. The public gearbox data set [34] is a data set consisting of vibration data recorded using Spectraquest’s gearbox fault diagnostic simulator. It includes healthy and broken tooth data and has been recorded under the variation of a load from “0” to “90” percent load with four different sensors in four directions. Ten text files are available for each case. The aim was to classify the data between the gearbox’s healthy and broken tooth conditions. We gained experience with this first step to adapt the DCNN to the needs of our data and the experimental setup. After that process, the topology of the DCNN was migrated to use the data from our experimental setup.

With two convolutional layers, five and ten filters, respectively, for each layer, and 1 × 20 as the filter size, the accuracy achieved by the 1D DCNN was around 70% for models trained with data from single sensors and 80% after the data fusion. To improve the results, we gradually added convolutional layers with an increasing number of filters and filter size at each step; the models improved 10% for some and 9% for others. Next, we added batch normalization layers and the nonlinear function ReLU, which performs a threshold operation on each element. The softmax activation function is used after the last fully connected layer to predict the output. We then reached an accuracy of around 100% in all cases, and we had a suitable set of parameters for the DCNN model.

The same scenario was performed with the LSTM method, i.e., training the model on a public data set and then using the resulting configuration to train the model on our data set. Unlike the DCNN model, the LSTM did not give convincing results with our data. This motivated the choice of the third method, CNN-LSTM, which is a combination of the two methods mentioned above.

**Figure 8:**
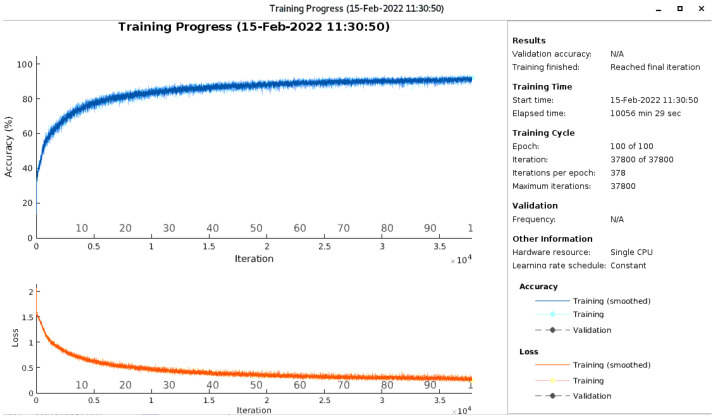
CNN-LSTM training progress.

The training of the third model was performed from scratch and was progressively improved according to the observations. For example, the gradient threshold, which was set to 1 for the LSTM, was set to infinity for the CNN-LSTM because during training, we realized that the gradient threshold could exceed the set limit, thus disturbing the learning process. At this stage, the fixed threshold control method was used to stabilize the process and speed up the learning. If the method is wrongly chosen, the model will not be effective in the end. However, by setting the gradient threshold to infinity, a significant performance improvement was achieved, with a huge training time as a counterpart. From one training of the model to the next, the number of epochs increased from 60 to 100 via 80 because the learning curve had not yet reached its peak (see Figure 8).

For all the methods, the global data set was randomly split; a total of 70% of the data samples were used as the training data and 30% as the test data. The models were developed using MATLAB on a Linux SMP server with a 48-core CPU and 64 GB RAM. The following section shows the results.

## 8. Results and Discussion

The experiments carried out in this work aimed at analyzing the sensor data for fault detection on the BLDC motor, materialized here by a set of variant shapes. Using three different methods, the sensor data were evaluated both individually and merged. Several metrics are available to evaluate the inference of the ML model. Here, Cohen’s Kappa (K), precision, recall, and accuracy were used and are defined according to the following Equations (1)–(4), where $P_{o}$: observed proportion correct, $P_{e}$: proportion expected correct, *T*: true, *F*: false, *N*: negative, *P*: positive.

(1) $$k=\frac{P_{o}-P_{e}}{1-P_{e}}$$

(2) $$Precision=\frac{TP}{TP+FP}$$

(3) $$Recall=\frac{TP}{TP+FN}$$

(4) $$Accuracy=\frac{TP+TN}{TP+FP+TN+FN}$$

### 8.1. Results of Models Trained on Single Sensor Data

**Figure 9:**
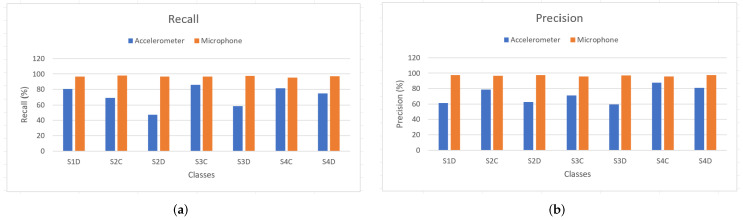
The DCNN models inferences in terms of recall and precision. (**a**) Recall. (**b**) Precision.

**Figure 10:**
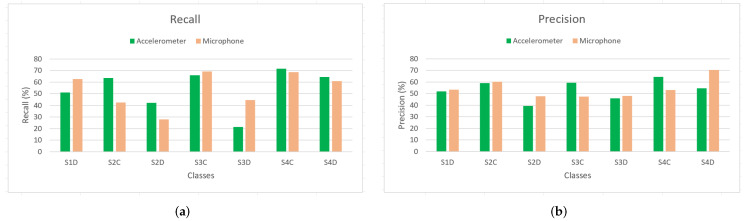
The LSTM models inferences in terms of recall and precision. (**a**) Recall. (**b**) Precision.

The DCNN and LSTM models were used to assess single sensor data. Figure 9 and Figure 10 show diagrams of the precision and the recall of the DCNN and the LSTM models respectively, to identify each shape using data from each sensor. Table 2 shows the overall accuracies of the models trained with single sensor data.

### 8.2. Results of Models Trained on Fused Sensor Data

**Figure 11:**
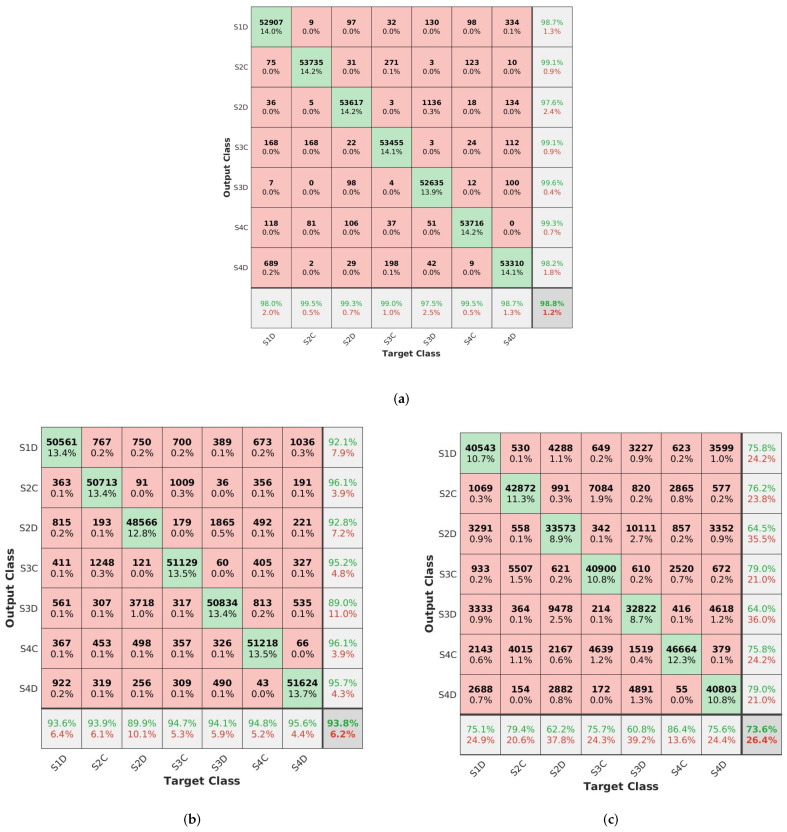
Confusion matrices of models trained with fused data. (**a**) DCNN confmat. (**b**) CNN-LSTM confmat (**c**) LSTM confmat.

As mentioned earlier, all the methods were used to train on fused signals data. To assess the resulting models, multi-class confusion matrices were computed and Cohen’s Kappa (K) was measured according to Equation (1). See the confusion matrices in Figure 11, where the column at the far right of the plot shows the precision, the row at the bottom of the plot shows the recall, and the cell at the bottom right of the plot shows the overall accuracy. The Cohen’s Kappa scores (k in %) obtained were as follows k = 98%, k = 92.79%, and k = 69.97% for the DCNN, the CNN-LSTM, and the LSTM, respectively.

### 8.3. Discussion

From different sensors, one can record particular measurements sensitive to varying types of conditions. This is why in our configuration, models trained with sounds data achieved different and better results than those trained with vibration data. These results also show that some sensors can be used individually, with an appropriate training model, as is the case for the DCNN model, which gave an accuracy of over 90% with the microphone data (see Table 2).

From the confusion matrices in Figure 11, we can see the results of models trained with fused data showing that the DCNN was more accurate than the CNN-LSTM and LSTM. The CNN-LSTM method achieved an accuracy of 93.8% compared to 73.6% for the LSTM method, which allowed us to confirm the hypothesis that the use of a convolutional layer in combination with LSTM could have a major impact in improving the performance of LSTM, which was the main reason for choosing the CNN-LSTM method. Although the models were trained on raw data without a feature extraction step, the ability of the DCNN to automatically learn relevant features from raw input signals is behind its good-quality results. The results in [35] indicated that the proposed DCNN-based fusion method also achieved the best result among other methods with around 98% accuracy on bearing fault diagnosis. The confusion matrices also present the amount of correctly and misclassified samples. We can easily observe that the DCNN was more robust and generalized better than the two other models since it registered just a few misclassifications over all the classes.

The impact of data fusion was most evident in the case of LSTM with around a 10% improvement over 2% for DCNN. Note that the maximum gain the DCNN could obtain was 4% because the baseline result was already very high (96%). Sensor data fusion is useful, even when it leads to only small improvements in some cases. The impact of the fusion can even vary during the operation of the setup. This means that a small impact in the average of the preciseness to predict wear out can lead in the specific case to a remarkable benefit. Additionally, small increases in preciseness can be of great value when a large machine can be prevented from becoming damaged.

## 9. Conclusions and Future Work

In this work, we examined the impact of data-level fusion on the accuracy of detecting different tools realized as load shapes on the BLDC motor. Such detection will be useful in the context of PM, where the detected shape in real machines, e.g., drills and cutter heads, can indicate the system status. We used a microphone and an accelerometer to record the corresponding signals under seven different load configurations. We built domain-specific DCNN, LSTM, and CNN-LSTM models to know which sensor provides the most useful information or if we can have a better set of information with a combination of sensors. The experiment conducted in this work also presented methods that can be applied in association with data-level fusion to achieve promising and good results in fault diagnosis on BLDC motors.

The microphone sensor data gave better accuracy than the accelerometer data, with an accuracy of 96.8% for the DCNN and 53.8% for LSTM. The combination of the two sensors showed an improvement of 2% with the DCNN and at least 10% with the LSTM. These models achieved an accuracy of 98.8% and 73.6%, respectively. The CNN-LSTM achieved an accuracy of 93.5% with the merged data. These results indicate that the DCNN can easily use data-level fusion to evaluate different BLDC engine states with different tools as equipment. The LSTM method did not achieve very interesting results by itself, but combined with a convolutional layer, the accuracy increased significantly. This means that the LSTM method has to be used with a feature extraction step to obtain the best results, as it cannot extract relevant features by itself for learning.

Motivated by the promising results of this work, in the future, we will investigate multi-sensor data fusion with different sensor positions and additional noise to diagnose the impact of the environment on the detection task. Additional sensors could be integrated. This could lead to the selection of an appropriate range of sensors for this particular purpose. As we have presented this work in the context of PM, it would be interesting in the future to connect the sensors to a small controller, which will transfer the data to a central node on which the sensing solutions will be deployed, which will evaluate the data directly. We also plan to extend the CNN-LSTM architecture used in this work to predict future failures based on the remaining useful life of the tool.

## Figures and Tables

**Table 1:** Data set.

Classes	Data Types	Number of Signals	Signals Length (Number of Points)
Class i; i = 1:7	Vibration	6	20,000,000
	Sound	6	20,000,000

**Table 2:** Accuracy of models trained with single sensor data.

	Accelerometer	Microphone
DCNN	71.1%	96.8%
LSTM	64.3%	63.8%

## Data Availability

The data presented in this study are available upon request from the corresponding author. The data are not yet publicly available as this is a new data set recorded during this study.

## Abbreviations

The following abbreviations are used in this manuscript:

BLDC	Brushless direct current
DCNN	Deep convolutional neural networks
LSTM	Long short-term memory
PM	Predictive maintenance
HW	Hardware
SW	Software
FPGA	Field-programmable gate array
PC	Personal computer
CNN-LSTM	Convolutional long short-term memory

## References

[1] Zhao J., Gao C., Tang T. (2022). A Review of Sustainable Maintenance Strategies for Single Component and Multicomponent Equipment. Sustainability.

[2] Liu Z., Meyendorf N., Mrad N. (2018). The role of data fusion in predictive maintenance using digital twin. AIP Conf. Proc..

[3] Jamil F., Jaradat M.A., Gruyer D., Najjaran H. (2020). Deep Learning Sensor Fusion for Autonomous Vehicle Perception and Localization: A Review. Sensors.

[4] Shifat T.A., Hur J.-W. (2021). ANN Assisted Multi Sensor Information Fusion for BLDC Motor Fault Diagnosis. IEEE Access.

[5] Gang Q., Siliang L., Donghui P., Huasong T., Yongbin L., Qunjing W. (2019). Edge computing: A promising framework for real-time fault diagnosis and dynamic control of rotating machines using multi-sensor data. IEEE Sens. J..

[6] Wathiq A., Sanjay S., Sutton R., Motwani A. (2015). A robust bearing fault detection and diagnosis technique for brushless DC motors under non-stationary operating conditions. J. Control. Autom. Electr. Syst..

[7] Shifat T.A., Hur J.-W. (2020). An effective stator fault diagnosis framework of BLDC motor based on vibration and current signals. IEEE Access.

[8] Ahmad W., Khan S.A., Islam M., Jong-Myon K. (2019). A reliable technique for remaining useful life estimation of rolling element bearings using dynamic regression models. Reliab. Eng. Syst. Saf..

[9] Nijat M., Lahann J., Emrich A., David E., Fettke P., Loos P. (2017). Time series classification using deep learning for process planning: A case from the process industry. Procedia Comput. Sci..

[10] Kästner F., Hübner M., Ohrem J., Clusserath L. Towards adaptive and efficient bottling plants in a cyber physical production system environment. Proceedings of the 2017 NASA/ESA Conference on Adaptive Hardware and Systems (AHS).

[11] Krüger J., Lehr J., Schlüter M., Bischoff N. (2019). Deep learning for part identification based on inherent features. CIRP Ann..

[12] Jiang K., Zhaohui J., Yongfang X., Zhipeng C., Dong P., Weihua G. (2020). Classification of silicon content variation trend based on fusion of multilevel features in blast furnace ironmaking. Inf. Sci..

[13] Serin G., Sener B., Ozbayoglu A., Unver H. (2020). Review of tool condition monitoring in machining and opportunities for deep learning. Int. J. Adv. Manuf. Technol..

[14] Ren L.C., Chia C.C., Chi L.C. (2011). Multisensor fusion and integration: Theories, applications, and its perspectives. IEEE Sens J..

[15] Luyang J., Taiyong W., Ming Z., Peng W. (2017). An Adaptive Multi-Sensor Data Fusion Method Based on Deep Convolutional Neural Networks for Fault Diagnosis of Planetary Gearbox. Sensors.

[16] Xiaohan C., Beike Z., Dong G. (2020). Bearing fault diagnosis base on multi-scale CNN and LSTM model. J. Intell. Manuf..

[17] Chen Z., Li W. (2017). Multisensor Feature Fusion for Bearing Fault Diagnosis Using Sparse Autoencoder and Deep Belief Network. IEEE Trans. Instrum. Meas..

[18] Jie L., Youmin H., Yan W., Bo W., Jikai F., Zhongxu H. (2018). An integrated multi-sensor fusion-based deep feature learning approach for rotating machinery diagnosis. Meas. Sci. Technol..

[19] Yuxuan C., Yi J., Galantu J. (2018). Predicting tool wear with multi-sensor data using deep belief networks. Int. J. Adv. Manuf. Technol..

[20] Li J., Yue J., Yang Y., Zhan X., Zhao L. (2017). Multi-Resolution Feature Fusion model for coal rock burst hazard recognition based on Acoustic Emission data. Measurement.

[21] Krishnakumar P., Rameshkumar K., Ramachandran K.I. (2018). Feature level fusion of vibration and acoustic emission signals in tool condition monitoring using machine learning classifiers. Int. J. Progn. Health Manag..

[22] Mönks U., Dörksen H., Volker L., Hübner M. (2016). Information fusion of conflicting input data. Sensors.

[23] Ouadah A., Zemmouchi-Ghomari L., Salhi N. (2022). Selecting an appropriate supervised machine learning algorithm for predictive maintenance. Int. J. Adv. Manuf. Technol..

[24] Ran Y., Zhou X., Lin P., Wen Y., Deng R. (2019). A Survey of Predictive Maintenance: Systems, Purposes and Approaches. arXiv.

[25] Serradilla O., Zugasti E., Rodriguez J., Zurutuza U. (2022). Deep learning models for predictive maintenance: A survey, comparison, challenges and prospects. Appl. Intell..

[26] Muhammad G., Alshehri F., Karray F., Saddik A.E., Alsulaiman M., Falk T.H. (2021). A comprehensive survey on multimodal medical signals fusion for smart healthcare systems. Inf. Fusion.

[27] Krishnamurthi R., Kumar A., Gopinathan D., Nayyar A., Qureshi B. (2020). An Overview of IoT Sensor Data Processing, Fusion, and Analysis Techniques. Sensors.

[28] Mahlisch M., Schweiger R., Ritter W., Dietmayers K. Sensorfusion using spatio-temporal aligned video and lidar for improved vehicle detection. Proceedings of the 2006 IEEE Intelligent Vehicles Symposium.

[29] Kozłowski E., Mazurkiewicz D., Żabiński T., Prucnal S., Sęp J. (2020). Machining sensor data management for operation-level predictive model. Expert Syst. Appl..

[30] Gamazo-Real J.C., Vázquez-Sánchez E., Gómez-Gil J. (2010). Position and speed control of brushless DC motors using sensorless techniques and application trends. Sensors.

[31] Mones Z. MEMS Accelerometer Based Condition Monitoring and Fault Detection for Induction Motor. Proceedings of the 7th International Conference on Engineering & MIS 2021.

[32] Kingma D.P., Ba J. (2017). Adam: A Method for Stochastic Optimization. arXiv.

[33] Wang S., Clark R., Wen H., Trigoni N. (2018). End-to-End, Sequence-to-Sequence Probabilistic Visual Odometry through Deep Neural Networks. Int. J. Robot. Res..

[34] Wang F., Zhang L., Zhang B., Zhang Y., He L. Development of Wind Turbine Gearbox Data Analysis and Fault Diagnosis System. Proceedings of the 2011 Asia-Pacific Power and Energy Engineering Conference.

[35] Wang X., Mao D., Li X. (2021). Bearing fault diagnosis based on vibro-acoustic data fusion and 1D-CNN network. Measurement.

